# Specialists Versus Trainees: Long-Term Secondary Deformity After Unilateral Cleft Lip Repair

**DOI:** 10.7759/cureus.70626

**Published:** 2024-10-01

**Authors:** Muath Mamdouh Mahmod Al-Chalabi, Wan Azman Wan Sulaiman

**Affiliations:** 1 Reconstructive Sciences Unit, Universiti Sains Malaysia (USM), Kota Bharu, MYS

**Keywords:** cleft surgery outcome, long-term cleft surgery outcome, plastic surgery training, secondary cleft deformity, surgical trainee

## Abstract

Background: Unilateral cleft lip repair is a common procedure in plastic and reconstructive surgery to restore both function and aesthetics. The long-term outcomes of these surgeries can be influenced by various factors, including the experience and position of the surgeon performing the procedure. This study aims to investigate whether there is a significant difference in long-term secondary deformities based on whether the surgery was performed by a specialist or a trainee.

Methods and materials: This is a retrospective cross-sectional study among unilateral cleft lip patients based on their data records. The study aims to assess the long-term secondary deformity in relation to the position of the surgeon (specialist or trainee) in patients who underwent unilateral cleft lip repair surgery at the Reconstructive Sciences Unit at Hospital Universiti Sains Malaysia (HUSM).

Results: Of the 50 patients, the majority (74%) were operated on by a specialist with more than five years of experience. Only 13 patients (26%) were operated on by trainees in the plastic and reconstructive surgery training program. Long-term secondary deformity had no significant relationship with the surgeon's position (chi-squared (χ^2^)(1) = 5.89, p = 0.08).

Conclusion: Long-term secondary deformities were less likely following unilateral cleft lip repair performed by a specialist or senior surgeon. However, the relationship between these secondary deformities and the surgeon's position (specialist or trainee) was statistically insignificant. Therefore, this study urges trainees to be more involved in simple cleft surgeries to enhance their surgical skills and achieve the best outcomes.

## Introduction

The most challenging aspect of cleft lip repair is achieving satisfactory postoperative results, especially in the long term when facial bone growth is complete and determines the ultimate facial appearance. Consequently, the treatment of clefts involves not only the surgical closure of the cleft but also achieving an aesthetically and functionally perfect result in adulthood [1,2]. The facial region where this deformity occurs is a highly visible body part, making the aesthetic and functional outcomes critically important [3].

Although surgical management typically falls under the purview of specialist doctors, the active involvement of trainees in such procedures is inevitable, particularly in teaching hospitals. In Malaysia, nearly all training programs are run by universities, and residents must complete four years of training before being registered as specialists. Surgical training is based on a practice model in which trainees are given opportunities to perform both supervised and unsupervised operations. While cleft surgeries are usually performed by specialists or senior surgeons, trainees still handle some cases, especially simpler cleft repairs.

Some reports have concluded that the mortality and morbidity rates of trainee-performed operations are higher, prompting some centers to evaluate this issue by comparing the functional outcomes of patients operated on by specialist doctors to trainees [4]. However, a literature search revealed no comprehensive reports evaluating the presence of secondary deformities following unilateral cleft lip repair in relation to the surgeon's position (specialist or trainee). Therefore, this study was designed to assess the significance of long-term secondary deformities concerning the surgeon's role to determine whether the surgeon's position influenced the outcome. This research aligns with modern treatment goals, which aim to minimize the number and impact of deformities associated with surgical intervention and to achieve the best possible surgical outcomes.

In this study, we assess the long-term results after the age of 14, as the midfacial projection reaches maturity at 14 years in males and 13 years in females [5]. Additionally, the cases included in this study were limited to those involving unilateral cleft lip repair. This focus was chosen because other types of clefts are typically not operated on by trainees in the plastic surgery training program, whereas trainees do have opportunities to perform unilateral cleft lip repairs.

## Materials and methods

This retrospective cross-sectional study assesses long-term outcomes in non-syndromic patients who underwent unilateral cleft lip repair within the first two years of life at the Reconstructive Sciences Unit at Hospital Universiti Sains Malaysia (HUSM) and whose current age is 14 years or older. The patients with incomplete medical records or those who underwent lip repair at other facilities but are receiving follow-up care at HUSM were excluded from the study. The study was conducted at the Reconstructive Sciences Unit at HUSM, a specialized center with expertise in cleft lip and palate surgery. Data collection spanned a defined period to ensure uniformity among patients treated within a specific age range.

Patient data were retrieved from medical records, which included demographic information (such as age, gender, and ethnicity), cleft characteristics (type and severity), details of the surgical procedure, and long-term secondary deformities. Specific emphasis was placed on the surgeon's post and experience level to determine their relationship to the development of secondary deformities. To investigate the impact of surgical expertise on outcomes, surgeons were classified into two categories: specialists and trainees. The aim was to assess whether the experience of the surgeon influenced the incidence of postoperative complications and long-term outcomes following cleft lip repair.

The analysis focused on comparing outcomes between patients treated by specialists and those operated on by trainees under the supervision of specialists. This comparative approach allowed for a thorough examination of whether the level of surgical experience played a role in influencing key postoperative variables, such as lip and nasal symmetry, scar formation, and functional impairments. The study aimed to provide insights into the potential role of surgeon experience in mitigating long-term deformities, thereby improving the standard of care in cleft lip repairs.

All data were entered into a structured proforma for consistency and accuracy during data collection. Following this, the data were analyzed using version 24 of the Statistical Package for Social Sciences (SPSS) (IBM SPSS Statistics, Armonk, NY). Descriptive statistics were used to summarize the demographic and clinical characteristics of the patients. Categorical variables were expressed as frequencies and percentages.

Ethical approval was obtained from the Institutional Review Board (IRB) of Universiti Sains Malaysia (approval number: USM/JEPeM/21120775). Due to the retrospective design of the study, the need for patient consent was waived by the ethics committee. However, strict confidentiality was maintained, and patient anonymity was preserved throughout the research process. Access to archived medical records was limited to authorized personnel only.

## Results

A total of 190 patients underwent unilateral cleft lip surgery between the years 1997 and 2008; however, only 50 patients were eligible to be included in this study. Their data records were accessed, reviewed, and analyzed.

Among the 50 patients included in this study, 37 (74%) were female, and 13 (26%) were male. The vast majority of patients (74%) were operated on by a specialist with more than five years of experience. Only 13 patients (26%) were operated on by trainees in the plastic and reconstructive surgery training program. The secondary deformities post-surgical repair of the cleft lip varied, affecting either the nose only, the lip only, or both. However, 13 patients (26%) showed no secondary deformity (Table 1).

**Table 1:** Demographic details of the patients

Variable	Frequency	%
Gender		
Male	13	26
Female	37	74
Post of surgeon		
Trainee	13	26
Specialist	37	74
Presence of secondary deformity		
None	13	26
Lip	9	18
Nose	13	26
Both	15	30

A chi-squared (χ^2^) test was used to investigate the relationship between the surgeon's post and the presence of secondary deformities. The presence of secondary deformity had no significant relationship with the surgeon's post (χ^2^(1) = 5.89, p = 0.08) (Table 2).

**Table 2:** Relationship between the surgeon's post and the presence of secondary deformity χ^2^: chi-squared

Post of the surgeon	Presence of secondary deformity		χ^2^	P
	No	Yes		
Trainee	1	12	5.888	0.08
Specialist	12	25		

## Discussion

Secondary deformities are common in children born with a cleft lip and palate. Correcting these deformities is an essential part of the care for those who have developed post-repair deformities [6]. In their study, Zhang et al. reported a 9.6% incidence of secondary lip deformities after lip repair [7]. In comparison, Cheema and Asim reported a 13.5% incidence, while we reported a slightly higher percentage of 18% [8]. However, our data analysis revealed that a quarter of the patients did not develop any secondary deformities. In contrast, 30% developed both lip and secondary nasal deformities, and 26% developed only secondary nasal deformities. This result suggests almost equal chances among these four outcomes.

The surgeon's post may influence the likelihood of secondary deformities. This was clearly observed in our study, which showed a higher proportion of patients with no secondary deformities who were operated on by a specialist (48%), compared to those operated on by a trainee (7.7%). We conducted a chi-squared test to evaluate the significance of the association between secondary deformities and the surgeon's position, but our results showed no significant relationship. This result is encouraging, as it suggests an equal possibility of secondary deformities after surgical repair by specialists or trainees. An extensive literature search was conducted to find similar studies discussing the surgical outcomes of unilateral cleft lip repair in relation to the surgeon's post, but none were found. However, as this is a concern in most surgical fields, we did find a few studies comparing the outcomes of surgeries performed by consultants, specialists, and trainees.

Lederhuber et al. conducted a study to assess the impact of trainee participation on inguinal hernia repair outcomes [9]. They reported that anterior mesh repairs for primary inguinal hernias in males are at higher risk of reoperation due to recurrence if a surgical trainee is the primary surgeon. Nevertheless, they concluded that this should not lead to trainees being barred from performing procedures but, rather, they should be supervised to improve their skills and increase their experience, which will shorten operation times and reduce complication rates.

In another study regarding the involvement of surgical trainees in colorectal cancer surgery and its effect on outcomes, Borowski et al. concluded that there was no difference in anastomotic leak rates, operative mortality, or survival between unsupervised trainees, supervised trainees, and consultants [10]. Kelly et al. published a meta-analysis comparing short-term oncological outcomes following colorectal resection performed by surgical trainees and expert surgeons [11]. They reported no difference between trainees and consultants in laparoscopic resection, local recurrence rates, or cancer-specific survival.

In Finland, Jokinen et al. compared hysterectomy outcomes between trainees and specialists [12]. They found that although trainees needed more time to perform hysterectomies, there were no differences between the trainees and the specialists with regard to significant complication rates.

On the other hand, Stranjalis reported that the outcome of cerebral aneurysm surgery, when adequately supervised, is not significantly affected by the neurosurgeon's seniority [4]. Therefore, Butterworth et al. suggested that training should be appropriate to the trainee's level and introduced with close supervision [13]. Schoenbrunner et al. investigated the effect of craniofacial fellowship training on cleft palate complication rates in a retrospective review [14]. They conclude that craniofacial fellowship training is associated with fewer postoperative fistulas. Similarly, Rintala and Haapanen investigated the correlation between the training and skill of the surgeon and the reoperation rate for persistent cleft palate speech [15]. They concluded that as the residents became specialists, their improvement varied greatly. Thus, both training and skill are essential for the patient's speech outcomes, but they are not necessarily synonymous terms.

## Conclusions

Although our data showed that long-term secondary deformities were less common after unilateral cleft lip repair when performed by a specialist, the association between secondary deformities and a surgeon's post (specialist or trainee) was statistically insignificant. Therefore, this study encourages trainees to have more opportunities in supervised operations, especially in simple cleft cases, to refine their surgical skills, ultimately leading to the best outcomes. Additionally, it encourages senior surgeons (consultants and specialists) to provide more opportunities for trainees.
